# School Boards and Medical Certificates

**Published:** 1898-04-23

**Authors:** 


### SCHOOL BOARDS AND MEDICAL CERTIFICATES.

It takes a good deal of time, no doubt, but it would be well for the profession, and highly conducive to its interests, if in every town medical men would take the trouble to concern themselves with public affairs and plant themselves in every public body to which they can obtain access. In many a village and small town the doctor is really the only representative of science, and even in towns of more pretentious character he may be one of a very select few in whose brains lies knowledge which might, if it were available, be of the greatest service to their fellow townsmen. Yet from a professional hesitation to appear pushing, from a dislike to "politics" and all its ways, and often no doubt from mere shyness and want of confidence in the readiness of their tongues, medical men are too apt to stand aside from all participation in local affairs, greatly, be it said, to the detriment of the districts in which they live.

As an instance of the advantage which the holding of public appointments by medical men may be both to their fellow citizens and to their profession, we would instance a matter which lately arose at a meeting of the West Bromwich School Board in regard to the long-vexed question of medical certificates. In this case Mr. Somers, a medical practitioner, moved the following resolution: "That having regard to the fact that the parents or guardians of a child unable to attend school through illness comply with their legal obligations by furnishing a certificate to that effect signed by any duly qualified medical practitioner, this Board considers such certificate as sufficient evidence of a child's inability to attend." In the end this resolution was carried, and so a very satisfactory solution was arrived at of a question which still troubles many Boards. In fact, it is the only satisfactory solution No doubt here and there a shaky certificate will come in, and if it does, and if it becomes clear that certificates are being given which are not true, by all means let the doctors be prosecuted. The medical profession will never sanction the giving of false certificates. But what is quite clear to us, and to all who have watched this question, is that the final decision as to a child's fitness for school so far as health is concerned, whether it be arrived at by a court of law, or by some special medical officer, or by any other means, must always be a medical affair, arrived at on medical evidence, and certified to by a medical man. Far better then to accept medical certificates at once, and so save the parents all the worry (keeping up the standard of the certificates by punishing all who give them on insufficient grounds, if such a thing ever happens) rather than leave the matter in the state of uncertainty in which it at present lies in certain districts, where no parent of a sick child can feel safe from prosecution owing to the doubt which exists as to whether or no the doctor's certificate will be accepted.

